# Co-lateralized bilingual mechanisms for reading in single and dual language contexts: evidence from visual half-field processing of action words in proficient bilinguals

**DOI:** 10.3389/fpsyg.2015.01159

**Published:** 2015-08-07

**Authors:** Marlena Krefta, Bartosz Michałowski, Jacek Kowalczyk, Gregory Króliczak

**Affiliations:** ^1^Action and Cognition Laboratory, Department of Social Sciences, Institute of Psychology, Adam Mickiewicz University in PoznańPoznań, Poland; ^2^Faculty of English, Adam Mickiewicz University in PoznańPoznań, Poland

**Keywords:** bilingualism, language context, overt reading, visual half fields, lateralization

## Abstract

When reading, proficient bilinguals seem to engage the same cognitive circuits regardless of the language in use. Yet, whether or not such “bilingual” mechanisms would be lateralized in the same way in distinct—single or dual—language contexts is a question for debate. To fill this gap, we tested 18 highly proficient Polish (L1) —English (L2) childhood bilinguals whose task was to read aloud one of the two laterally presented action verbs, one stimulus per visual half field. While in the single-language blocks only L1 or L2 words were shown, in the subsequent mixed-language blocks words from both languages were concurrently displayed. All stimuli were presented for 217 ms followed by masks in which letters were replaced with hash marks. Since in non-simultaneous bilinguals the control of language, skilled actions (including reading), and representations of action concepts are typically left lateralized, the vast majority of our participants showed the expected, significant right visual field advantage for L1 and L2, both for accuracy and response times. The observed effects were nevertheless associated with substantial variability in the strength of the lateralization of the mechanisms involved. Moreover, although it could be predicted that participants' performance should be better in a single-language context, accuracy was significantly higher and response times were significantly shorter in a dual-language context, irrespective of the language tested. Finally, for both accuracy and response times, there were significant positive correlations between the laterality indices (LIs) of both languages independent of the context, with a significantly greater left-sided advantage for L1 vs. L2 in the mixed-language blocks, based on LIs calculated for response times. Thus, despite similar representations of the two languages in the bilingual brain, these results also point to the functional separation of L1 and L2 in the dual-language context.

## Introduction

In the majority of people, the left hemisphere is typically involved in the control of language and its many related skills. Yet a strength, and in some cases even a direction, of their laterality is often modulated by the actual linguistic experience, including the onset of exposure to different languages and the achieved fluency (e.g., Perani et al., 1998; Klein et al., 2006; Grossi et al., 2010). Indeed, the overall organization of languages in the human brain seems to depend on whether they are acquired simultaneously, or rather the non-native language(s) is (are) acquired later in life, with a degree to which the level of proficiency affects language laterality being a more debatable factor (for a meta-analysis of behavioral studies on bilingual language lateralization, see Hull and Vaid, 2007; for a targeted review of neuroimaging work on this topic, see Abutalebi, 2008).

While most of the studies on language laterality in the bilingual, or multilingual, brain have capitalized on selected aspects of language production (e.g., picture naming or other stimulus-driven word generation) or language comprehension (e.g., semantic categorization of the visually or aurally presented words), relatively little is known about the lateralization of *bilingual mechanisms* involved in such a highly automated linguistic skill as overt reading. Although there is evidence that when a person becomes equally proficient in two or more languages, skilled reading in each of them could engage largely the same neural areas or circuits involved in related mechanisms (cf. Meschyan and Hernandez, 2006; e.g., Parker Jones et al., 2011), this principle might be particularly relevant to situations where two languages either are, even if unintentionally, or must be available for task performance at the very same time (cf. Grosjean, 2001). Consequently, a question remains whether or not the same rule applies when one uses a single language at a given time, and there is neither need nor point to have the other language in readiness (for a brief review, see Wu and Thierry, 2010; see also Van Heuven and Dijkstra, 2010; Spalek et al., 2014).

To shed some light on this issue, we asked proficient Polish-English bilinguals to read aloud action words in one of the two languages alone or—in the later test—to read these same words in the dual-language context. Although such tasks seem quite basic for these two alphabetic scripts, they may still involve many of the left-lateralized mechanisms. This is definitely the case for simple graphic processing of visual word forms (which is typically carried out by the left cortical and subcortical structures, e.g., McCandliss et al., 2003; Cohen and Dehaene, 2004), but the engagement of the dominant hemisphere can be weakened at the level of phonological/semantic processing, depending on the language involved and the age of its acquisition (Leonard et al., 2010; Peng and Wang, 2011; see also Hull and Vaid, 2007). Notably, the relative contribution of the two hemispheres to overt reading should be easily revealed by the pattern of accuracy and/or response times to target words presented in one of the two visual half fields (VHFs). Indeed, when used properly, the method we adopted here is a very reliable measure of cerebral language dominance. Since the outcomes obtained this way have been shown to strongly correlate with neuroimaging results concerning language laterality (Hunter and Brysbaert, 2008), this method can be successfully used, as a much more economical alternative to the traditional methods, to assess the laterality of the two languages in question.

In sum, this study utilized a very simple but reliable test of language lateralization and applied it to a population of proficient bilinguals. We focused on one particular category of stimuli, i.e., action words, which typically engage concepts that are strongly left lateralized (for review, see Binkofski and Buxbaum, 2013). Therefore, any alleviation of the strength of their lateralized processing could point to a reorganization of the language circuits due to early acquisition of the second language. Moreover, the study involved two separate phases. In the first one, the testing procedures unambiguously pointed to one language only, whereas the second phase invoked the two languages simultaneously. As a result, reading in the single-language context in the VHF paradigm should unequivocally inform us about the laterality of each of the languages. The dual-language context, on the other hand, allowed us to resolve the issue of whether or not the earlier results concerning the laterality of a given language could be affected by the participants' adoption of an intermediate strategy to be equally efficient in both languages, or rather by the between-language interference (or lack of thereof) from the non-target visual field.

Because very proficient bilinguals were tested, we did not expect any differences in response accuracy between the two languages. Yet, if any between-language interference was present, it was more likely to occur in the non-dominant VHF, and possibly for the non-native language. Such effects were predicted unless participants adopted a truly intermediate strategy, which was likely in our highly proficient sample. Finally, given that two of our participants could potentially be classified as infant bilinguals, three others were really close to the adult bilingual category, and the remaining 13 started acquiring the second language between the ages of 7 and 10, we expected a large variability in the strength of the lateralization of their two languages (e.g., Hull and Vaid, 2007). Such variability is an asset (see Biduła and Króliczak, 2015), because it is paramount in testing for correlations between the laterality indices obtained for the two languages. They were of course expected to correlate quite strongly.

## Methods

The first author obtained a positive opinion about the to-be-used procedures and protocols from the local Ethics Committee for Research Involving Human Subjects. Carried out in *Action and Cognition Laboratory* in the Institute of Psychology at Adam Mickiewicz University in Poznań, Poland, the study conformed to the 2013 WMA Declaration of Helsinki.

### Participants

Eighteen healthy volunteers (16 women, age: 18–32, mean = 23.3, *SD* = 2.9) took part in the experiment after giving their written informed consent. All of them had normal or corrected-to-normal visual acuity. Fifteen individuals declared themselves as right-handers, and three as left-handers. All participants were native speakers of Polish (L1) who began to learn English (L2) as a foreign language between the ages of 5 and 11 (mean = 8.2, *SD* = 2.1). At the time of the experiment, all subjects were highly proficient users of both languages. Their fluency in L2 was established in two ways: on the basis of their field of study—English Philology at Adam Mickiewicz University in Poznań, Poland—and/or the language certificates obtained by passing at some point of their studies standardized tests of English language proficiency, i.e., possessing at least the *Certificate in Advanced English* (CAE), or *International English Language Testing System* (IELTS) with the result of seven points or above.

### Stimuli

Forty Polish and 40 English verbs denoting manual activities that require the use of simple or complex tools were used as stimuli. All the activities were commonly known and frequently performed. This was established in an earlier pilot study, wherein eight individuals rated the familiarity of Polish and English words from a greater set on a scale of 1 (unfamiliar word) to 5 (very familiar word). Only words that received an average of 3 points or above were included in the experimental set. Care was taken to ensure that the verbs in both languages corresponded to each other in their meaning. The stimuli were in their infinitive form (Polish, English), or non-finite, gerund form (English). The rationale for the latter manipulation was to minimize the difference in length between Polish and English verbs, as Polish verbs are typically longer than the English ones. Ten English verbs were kept in their infinitive form to match the shortest Polish verbs. The two sets of words did not differ significantly in terms of the average word length [*t*_(78)_ = 0.88, *p* = 0.38]. The number of words starting with voiced or voiceless initial phoneme was the same for both languages, with 18 words beginning with a voiced phoneme and 22 with a voiceless one. For the list of stimuli used in the experiment, see Appendix 1 in Supplementary Materials.

### Procedure

**Figure 1:**
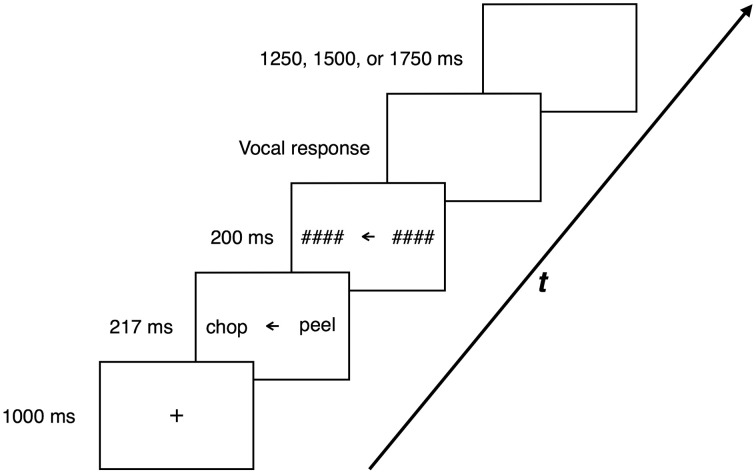
**Trial structure and timing**. After a fixation point presented on a blank screen for 1000 ms, two words (the target stimulus and the non-target stimulus) were shown bilaterally for 217 ms, with a central arrow pointing to the location of the target. The stimuli were then covered by 200-ms masks. After the onset of participant's vocal response, a blank screen of a variable duration (1250, 1500, or 1750 ms) was introduced and preceded the next trial.

Participants were seated in front of the screen at a viewing distance of ~57 cm. Each trial began with a central fixation cross of 1000-ms duration. Next, two words were presented in the left and right visual field with a central arrow pointing to the left or right. The role of the arrow was to indicate the target word. Participants were instructed to read the target word aloud, and to ignore the other, non-target word. All stimuli were presented on a white background in Arial font, color black, size 50 points, 2° of the visual angle from the central arrow. Although Hunter and Brysbaert (2008) suggested that in a VHF paradigm the stimuli should not be visible for more than 200 ms, our pilot study revealed that with the adopted parameters of the procedure and stimuli, average response accuracy in the dominant field was only about 70%. By using results from a 3-down-1-up staircase procedure, we adjusted the duration of the target stimulus to 217 ms in order to achieve accuracy of approximately 75% (cf. McNair and Harris, 2012). Thus, after 217 ms, both words were masked with strings of hash marks. The length of the presented string was always equal to the length of the masked word. Then, a blank screen appeared and stayed until a vocal response was registered. The response time, as measured by the onset of the vocal reaction (detected by the SV-1 Smart Voice Key: http://www.cedrus.com/sv1/), was recorded by the software used for stimulus presentation (SuperLab 4.5 by Cedrus: http://www.superlab.com/). The accuracy of the response was constantly monitored by the experimenter. A blank screen of variable (1250, 1500, or 1750 ms) duration was introduced between the successive trials. The trial structure is depicted in Figure 1.

Before the experiment proper, a training session consisting of two single-language blocks, each containing five trials, was administered. Words used during the training session did not appear in the subsequent experimental session. For each participant, the language of the first training block was the same as the language of the first single-language experimental block. The language of instructions always corresponded to the language used in a given block. In the dual-language blocks, the language of instructions was changed every consecutive sentence.

The experiment consisted of six blocks of pseudo-randomly presented trials. At the beginning of each block, participants were informed of its language and/or type (Polish single-language, English single-language, or mixed-language). In the four single-language blocks (two Polish blocks, and two English blocks, 40 trials in each), two words presented in every trial came from the same language (Polish, or English, respectively). In the two mixed-language blocks (80 trials in each), the target word came from one language, and the non-target word came from the other one. In both types of blocks, the primary criterion of assigning words into target—non-target pairs was their length. Each of the eighty stimulus words was presented as a target only four times: two times in single-language blocks (once in the LVF, and once in the RVF), and two times in mixed-language blocks (again, once in the LVF, and once in the RVF). Moreover, in the whole experiment, every word was presented four times as a non-target stimulus. As a result, there were a few trials in which the presented words differed in length by no more than two characters. Mixed-language blocks were always presented as the last, whereas the order of single-language blocks (two consecutive Polish blocks, and two consecutive English blocks) was counterbalanced across participants.

### Statistical analyses

The pattern of performance (i.e., accuracy and response times) demonstrated by the three left-handed individuals closely resembled the outcomes of right-handed participants, which is in line with the observation that in the majority of left-handers, language skills are represented in a way similar to their representations in typical, right-handed subjects, at least in the case of simple verbal fluency tests (e.g., Knecht et al., 2000; Króliczak et al., 2011). Therefore, in order to increase statistical power, the results of all 18 participants were analyzed together. To this end, we used two separate repeated-measures Analyses of Variance (ANOVAs), one for accuracy and one for response times to correctly read words. The within-subjects factors were *block type* (single-language, mixed-language), *target language* (Polish, English), and *target location* (left, right). The adopted level of significance was α = 0.05. If necessary, the required *post-hoc* tests were Bonferroni corrected. Response times exceeding 2.5 s were removed due to the possibility of (1) participants guessing the answer, and/or (2) an equipment malfunction. Also, for reaction times accompanying correctly read words, outliers greater than two standard deviations above or below the mean (calculated for each condition) were removed. Consistent with Hunter and Brysbaert (2008), in such a difficult task and for different reasons (primarily incorrect or too long responses), an average of 34.8% trials for each participant were removed, with only 24.4% trials for target word presented on the right, and 45.2% of trials for target word presented on the left.

In order to determine the hemispheric dominance for the first (Polish) and second (English) language, lateralization indices (LIs) for both languages, within each context (single-language, dual-language), as well as across both tested contexts, were derived through the following formulas, separately for reading accuracy (LI_ACC_) and response times (LI_RT_):

$$\begin{array}{lll} {\text{LI}}_{\text{ACC}} & = & {\left[\left(\text{R}-\text{L}\right)∕\left(\text{R}+\text{L}\right)\right]}^{*}100\\ {\text{LI}}_{\text{RT}} & = & {\left[\left(\text{L}-\text{R}\right)∕\left(\text{L}+\text{R}\right)\right]}^{*}100 \end{array}$$

For LI_ACC_ calculations, R and L represent accuracy of reading words presented in the RVF and LVF, respectively, in the single-language context, in the dual-language context, or across both contexts. For LI_RT_ calculations, R and L represent response times (reading onsets) for words presented in the RVF and LVF, respectively, in the single-language context, in the dual-language context, or across both contexts. The obtained results allowed us to determine which visual half-field, and also indirectly which cerebral hemisphere, was the dominant one in the processing of Polish and English words for each participant. In the case of both LI_ACC_ and LI_RT_, positive values indicated right visual field/left hemisphere advantage in reading words of a given language, whereas negative values—left visual field/right hemisphere advantage in the task in question.

Finally, to investigate whether or not the representations of both L1 and L2 share any common organizational features, we performed a correlational analysis of the obtained LIs, as well as additional pairwise comparisons. Significant correlations between LIs for L1 and L2 in each of the contexts would indicate that the lateralization of the first and second language in highly-proficient bilinguals from our sample depends on one another, although they may not necessarily be similarly represented in the brains of the participants. A lack of correlations would suggest that these languages are represented independently, or even quite separately, even if they are not lateralized differently. On the other hand, significant differences obtained between LIs for L1 and L2 might indicate that one of the hemispheres is differently involved in the processing of words from each of these two languages.

All statistical analyses were carried out using SPSS 20.0 (SPSS Ins., Chicago, IL).

## Results

### Reading accuracy

**Figure 2:**
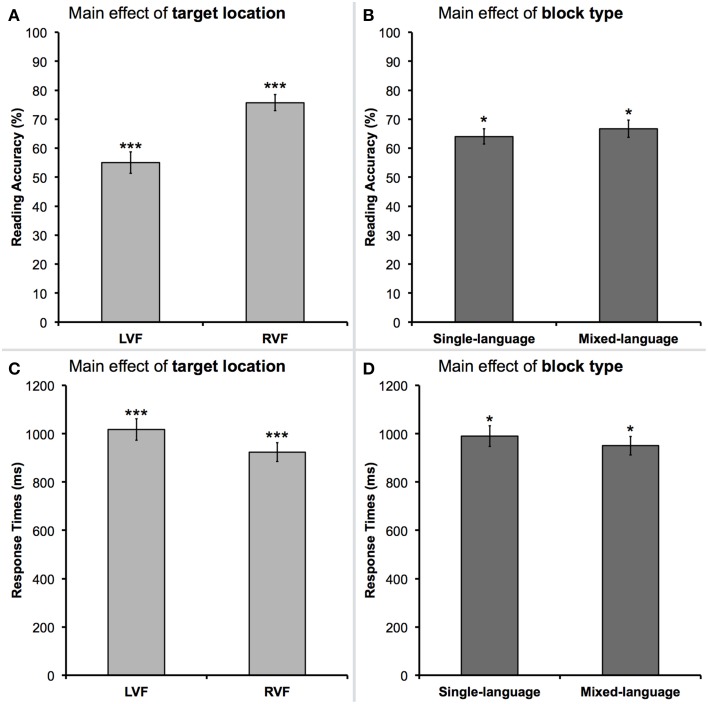
**The main effects of target location and block type**. **(A,B)** Depict the results for reading accuracy. **(C,D)** Depict the results of response times for correctly read words. Target words presented in the right visual field (RVF) were read with both greater accuracy and faster response times, as compared to words presented in the left visual field (LVF). In the mixed-language blocks, target words were read with both greater accuracy and faster response times, as compared to words presented in single-language blocks. Asterisks indicate a difference with *p*-value of 0.05 (^*^) or 0.001 (^***^).

There was a main effect of *target location* [*F*_(1, 17)_ = 33.6, *p* < 0.001, Partial Eta Squared (_*p*_η^2^) = 0.66], such that words presented in the RVF were read more accurately than words presented in the LVF [average reading accuracy in the RVF = 75.7%, standard error (*SE*) = 2.8% vs. LVF = 55.0%, *SE* = 3.7%]. This effect is shown in Figure 2A. We also observed a main effect of *block type* [*F*_(1, 17)_ = 4.6, *p* < 0.05, _*p*_η^2^ = 0.21], although quite counterintuitively the words in mixed-language blocks were read more accurately than words in single-language blocks (average accuracy of reading in mixed-language blocks = 66.7%, *SE* = 2.9% vs. single-language blocks = 64.0%, *SE* = 2.6%). This effect is depicted in Figure 2B. There was also a trend toward a main effect of *target language* [*F*_(1, 17)_ = 3.1, *p* = 0.10, _*p*_η^2^ = 0.16]. Namely, participants tended to read target words in Polish with greater accuracy as compared to words in English (average reading accuracy in Polish = 66.5%, *SE* = 2.8% vs. English = 64.2%, *SE* = 2.9%). No further significant effects were found, including the lack of clear trends toward interactions.

### Response times (RTs) for correctly read words

Similarly to the analysis of reading accuracy, the predicted main effect of *target location* [*F*_(1, 17)_ = 18.4, *p* < 0.001, _*p*_η^2^ = 0.52] was observed. Namely, for the correctly read words presented in the RVF, response times were significantly faster than for the correctly read words presented in the LVF (mean RT in the RVF = 923 ms, *SE* = 39 ms vs. LVF = 1017 ms, *SE* = 44 ms]. This effect is shown in Figure 2C. A main effect of *block type* [*F*_(1, 17)_ = 5.6, *p* < 0.05, _*p*_η^2^ = 0.25] revealed that participants took longer to read words in single-language blocks than in mixed-language blocks (mean RT for single-language blocks = 990 ms, *SE* = 43 ms vs. mixed-language blocks = 951 ms, *SE* = 38 ms). This effect is shown in Figure 2D. There was also a main effect of *target language* [*F*_(1, 17)_ = 11.4, *p* < 0.01, _*p*_η^2^ = 0.40], such that participants read words in Polish significantly faster than words in English (mean RT for Polish words = 947 ms, *SE* = 42 ms vs. English = 994 ms, *SE* = 39 ms). No other effects reached or even approached significance level. The mean RTs, as well as average accuracy data, for all the conditions are listed in Table 1.

**Table 1:** **Block type (single-language, mixed-language), target language (Polish, English), target location (Left Visual Field, LVF; Right Visual Field, RVF) with their mean response times (ms), accuracy (%), and their standard errors of the means**.

**Trial type**			**Response times (ms)**	**St. error**	**Accuracy (%)**	**St. error**	***N***
Single-language	Polish	LVF	1002	57	58.2	3.3	18
		RVF	918	39	74.2	3.1	18
	English	LVF	1070	50	50.8	3.7	18
		RVF	972	40	72.9	3.1	18
Mixed-language	Polish	LVF	990	43	55.0	4.4	18
		RVF	877	43	78.8	3.4	18
	English	LVF	1007	39	56.0	5.0	18
		RVF	928	39	77.1	2.9	18

### Laterality indices (LIs)

**Figure 3:**
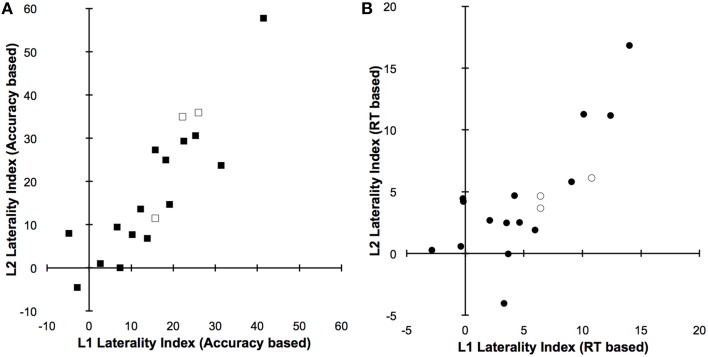
**Laterality indices (LIs) for Polish (L1) and English (L2) based on (A) reading accuracy and (B) response times regardless of the reading contexts**. There were significant correlations between L1 and L2 LIs, for both reading accuracy and response times. Positive scores indicate language representation lateralized more to the left hemisphere, negative scores to the right hemisphere, and 0 suggests that the representation is symmetrical between both hemispheres. Empty shapes indicate left-handed participants from our sample.

**Figure 4:**
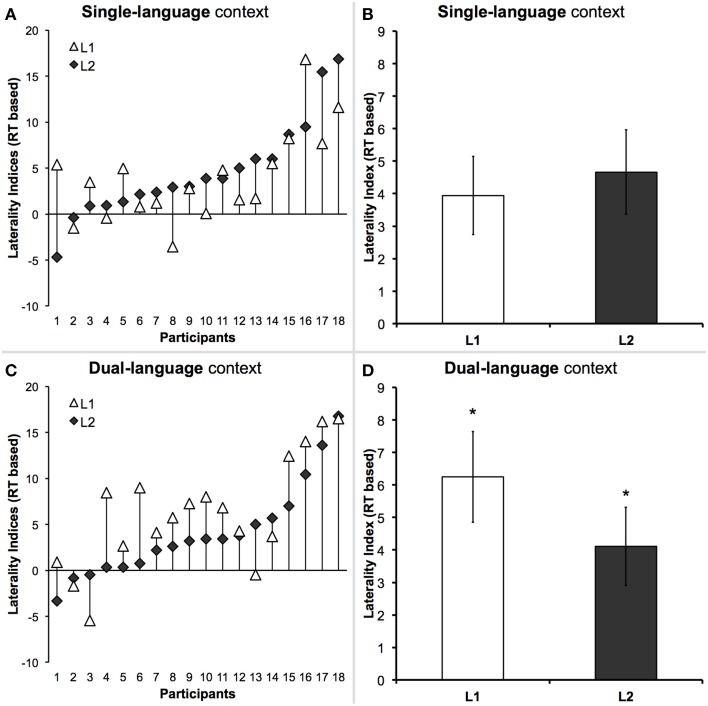
**Context-dependent laterality indices (LIs) for both languages based on response times (RTs)**. **(A)** Individual LIs for L1 (participants marked with white triangles) and L2 (black diamonds) in the single-language context. Participants were sorted in the ascending order according to their LIs for English. **(B)** A comparison of mean LIs from the single-language context. In the single-language context there was a significant correlation between LIs for both languages, accompanied by no significant difference between them. **(C)** Individual LIs for L1 (white triangles) and L2 (black diamonds) in the dual-language context. **(D)** A comparison of mean LIs from the dual-language context. In the dual-language context there was a clear reorganization of L1, such that, although there was still a significant correlation between LIs for both languages, there was also a significant difference between them. Notably, it was L1 (Polish) that was lateralized significantly more to the left hemisphere than L2 (English). Asterisks indicate a difference with *p*-value of 0.05 (^*^). For further details, see also Table 2.

The results of correlational analyses are shown in Table 2. As expected, we found strong significant correlations between individuals' Polish and English LIs, for both reading accuracy and response times, in single-language context, in dual-language context, as well as across both contexts. The latter effects are shown in Figures 3A,B. Importantly, in the single-language context there was no significant difference between RT-based LIs for both languages. Individual LIs for the single-language context are shown in Figure 4A, and mean LIs in Figure 4B. In the dual-language context, however, we observed a significant right visual-field/left hemispheric advantage for reading Polish, as compared to English, words [Polish *LI* = 6.2, *SE* = 1.4 vs. English *LI* = 4.1, *SE* = 1.2; *t*_(17)_ = 2.4, *p* < 0.05]. Individual LIs for the dual-language context are shown in Figure 4C, and mean LIs, as well as a significant difference between them, in Figure 4D.

**Table 2:** **The table shows the ***p***-values (and ***r***-values) of the correlations between the Laterality Indices (LIs) calculated for Polish and English within each of the experimental conditions (single-language, dual-language), as well as across them (general)**.

	**Polish, single**	**Polish, dual**	**Polish, general**	**English, single**	**English, dual**	**English, general**
**CORRELATIONS FOR READING ACCURACY LIs (LI_ACC_)**						
Polish, single	–	**0.18 (0.33)**	< 0.001 (0.73)	<**0.05 (0.53)**	< 0.05 (0.55)	< 0.01 (0.62)
Polish, dual	–	–	< 0.001 (0.88)	< 0.05 (0.57)	<**0.001 (0.80)**	< 0.001 (0.80)
Polish, general	–	–	–	< 0.01 (0.68)	< 0.001 (0.84)	<**0.001 (0.88)**
English, single	–	–	–	–	<**0.05 (0.52)**	< 0.001 (0.81)
English, dual	–	–	–	–	–	< 0.001 (0.92)
English, general	–	–	–	–	–	–
**CORRELATIONS FOR RESPONSE TIME LIs (LI_RT_)**						
Polish, single	–	**0.07 (0.44)**	< 0.001 (0.82)	<**0.01 (0.61)**	0.11 (0.39)	< 0.05 (0.56)
Polish, dual	–	–	< 0.001 (0.88)	< 0.05 (0.56)	**<0.001 (0.78)**	< 0.001 (0.73)
Polish, general	–	–	–	< 0.01 (0.68)	< 0.01 (0.70)	<**0.001 (0.76)**
English, single	–	–	–	–	<**0.01 (0.67)**	< 0.001 (0.92)
English, dual	–	–	–	–	–	< 0.001 (0.91)
English, general	–	–	–	–	–	–

*The upper part of the table reports the correlations between LIs calculated on the basis of reading accuracy, whereas the lower part reports the correlations between LIs calculated on the basis of response times. Pairs of LIs that were of particular interest are highlighted in bold. Additionally, shaded cell indicate the pair wherein LIs significantly differed from each other*.

### *Post-hoc* analyses and results for the exclusion of possible interpretations

To rule out the possibility that the differences between L1 and L2 reading latencies were caused by variations in voice-key sensitivity, we carried out a *post-hoc* analysis of the voicing of initial phonemes for the tested words. Voicing has been previously shown to affect the measured response times, with most voiced phonemes being detected faster than voiceless phonemes (Kessler et al., 2002). With this in mind, we ran a repeated-measures ANOVA for the frequencies (expressed in % correct) with which words from both languages were accurately read in each of the experimental conditions. The within-subjects factors were *block type* (single-language, mixed-language) and *target location* (left, right), whereas the between-subjects factors were *target language* (Polish, English) and *voicing* (voiced, voiceless). Neither the main effect of *voicing* [*F*_(1, 76)_ = 2.6, *p* = 0.11] nor any interactions including this factor were statistically significant.

Because the aforementioned analysis demonstrated that correct responses to voiced and voiceless phonemes were in fact distributed equally across different conditions therefore any differences with which they would be recorded by voice-key should not play a role. Consistent with such a hypothesis, except for the main effect of *voicing* [*F*_(1, 76)_ = 18.9, *p* < 0.001] such that reading onset of words starting with voiced phonemes was indeed detected significantly faster, (and the familiar main effect of side, such that words in the right visual field were read significantly faster than words in the left visual field), none of the remaining main effects, nor One- or Two-Way interactions even approached significance level, and a trend in the Four-Way interaction was completely irrelevant to the findings reported here.

To rule out the possibility that any effects observed in the final mixed-language blocks might be due to practice effects (e.g., Garofeanu et al., 2004), we ran two 4 (*block number*) × 2 (*target location*) repeated measures ANOVAs for accuracy and response times to correctly read words in single-language blocks. There was no evidence that participants' accuracy increased with practice in consecutive blocks, as revealed by no main effect of *block number* [*F*_(3, 51)_ = 0.5, *p* = 0.71, _*p*_η^2^ = 0.03]. In fact, after initial (non-significant, *p* = 0.32) improvement in the second block, accuracy in the last block decreased. Moreover, there was no evidence that participants' performance, as measured by response latencies, improved with practice in consecutive blocks. This was revealed by no main effect of *block number* [*F*_(3, 51)_ = 0.6, *p* = 0.61, _*p*_η^2^ = 0.04]. In fact, after initial (non-significant, *p* = 0.13) improvement (i.e., response time decrease) in the second block, response times increased in the subsequent blocks.

## Discussion

In this study we examined the lateralization pattern of overt word reading in single- and dual-language contexts in highly proficient Polish-English bilinguals. It was possible thanks to the utilization of the visual half-field paradigm in which in the single-language blocks only words from one language were presented and read, whereas in the mixed-language blocks words from both languages were presented and read.

Both for accuracy and for response times (or reading latencies), there was a greater advantage for reading words presented in the RVF, as opposed to the LVF. Such effects as superior accuracy of word processing and shorter response latencies that accompany a given task performed in the RVF—irrespective of the language in use—clearly indicate that the bilingual mechanisms involved in task performance both in L1 and L2 are predominantly lateralized to the left hemisphere. These results are consistent with the well-established findings that in the vast majority of people, irrespective of handedness, the number of languages acquired, and the bilingual (or even multilingual) status, language and its related skills, such as gestures, are typically represented in the left hemisphere or are at least mediated by critical left-lateralized mechanisms, including access to relevant concepts (e.g., Knecht et al., 2000; Vingerhoets et al., 2003; Króliczak et al., 2011; Vingerhoets et al., 2013; see also Króliczak, 2013; for review, see Hull and Vaid, 2007).

Despite high bilingual proficiency and the resulting lack of differences in L1 and L2 reading accuracy, the words in English were nonetheless read significantly slower than words in the native Polish. Of course, any simple differences between L1 and L2 in response times could be accounted for by the frequency of use of words from both languages in daily communication. Indeed, this interpretation is consistent with the findings that, unlike language proficiency, the daily pattern of bilingual language use is often not correlated with onset age of bilingualism (Flege et al., 2002), and may even be negatively correlated (Luk and Bialystok, 2013). In consequence, not only the activation of L2 phonology may be delayed (Spalek et al., 2014), but also the less rehearsed English may put greater motor demands on word articulation (cf. Parker Jones et al., 2011).

Counter to earlier reports suggesting that in comparison to other tongues, English is one of the most left-lateralized languages (e.g., Newman et al., 1999; Halsband, 2006), our results indicate that this is not always the case. Here, in the single-language context the two languages tested were similarly lateralized, whereas in the dual-language context it was the native Polish that showed greater left-sided laterality. Despite these differences, which were clearly dependent on the context, the laterality of both languages was nevertheless strongly correlated. Namely, the direction and strength of laterality for one language was always followed by a similar effect for the other, including the very rare reversed (right-sided) laterality for both. This observation is no doubt consistent with the idea that in a bilingual brain there are common mechanisms, perhaps at several different levels of language processing, that enable the fluent command of the acquired languages (e.g., Dijkstra and van Heuven, 2012).

As such, our results demonstrate that the visual half-field paradigm is not only a great method for measuring the lateralization of language, but can be equally effective in testing asymmetries of language processing in different contexts.

### The functional separation of L1 and L2 in the dual-language context

As demonstrated by no effect of language on reading accuracy, the tested group did consist of highly proficient bilinguals. To our surprise, for such individuals, reading in the single-language context was much harder than performing the same task in the dual-language context (cf. Canseco-Gonzalez et al., 2010; see also Cheng and Howard, 2008). Importantly, this effect is consistent with slower responses in the single-language context and, together, these results suggest greater within- than between-language interference, regardless of whether L1 or L2 is tested.

Although we hypothesized that the requirements for reading would increase in the dual-language context and, therefore, even if unintentionally, could lead to reliance on the same neural circuits, this was not the case. On the contrary, the most critical outcome of this study is the observation that despite the common direction of hemispheric asymmetries, as shown by strong correlations between the LIs for the two languages, their pattern undergoes a significant functional reorganization in the dual-language context. This outcome is consistent with earlier studies showing the effects of context in which a bilingual language user operates at a given time on task performance (e.g., Marian and Spivey, 2003a,b; Canseco-Gonzalez et al., 2010). Specifically, in the paradigm used here, in the single-language context the comparison of LIs for both languages did not reveal any differences in the strength of their asymmetry. Conversely, in the dual-language context, L1-related reading mechanisms were significantly more strongly lateralized to the left hemisphere than the mechanisms for reading in L2. Indeed, this unexpected shift, typically in the form of increased left-sided L1 laterality, was also somewhat unpredictable because when L1 performance in single- and dual-language contexts was compared, there were no significant correlations between LIs both for accuracy and for response times, whereas these correlations were still present for reading in L2.

These results strongly indicate that when two languages must be available at the same time the mechanisms involved in their control get functionally separated rather than merged. The consequence of such reorganization, either automatic or strategic, could be the minimization of the costs of maintaining readiness of the two languages and/or the increase of the efficacy of using them in parallel (cf. Christoffels et al., 2007; Cheng and Howard, 2008). This scenario—a separation of the mechanisms involved in lexical and/or phonological access—is way more likely than any reorganization of the laterality of tool-use concepts, which in the majority of individuals should still be strongly left-lateralized (Króliczak and Frey, 2009; Michałowski and Króliczak, 2015).

## Limitations of the study

The paradigm could benefit from the monitoring of eye movements, although the simultaneous bilateral presentation of the target and non-target words with an additional central cue controlling participants' attention should successfully prevent participants from making express saccades toward the target word when it is still visible. The immediate backward masking procedure, on the other hand, makes a regular saccade in that direction rather useless (Helon and Króliczak, 2014). Moreover, the inclusion of pseudowords as non-target stimuli could shed some new light on the possible within- and between-language interference effects observed and discussed here.

## Conclusions

All in all, this study convincingly demonstrates that the asymmetries of language processing in the bilingual brain can be effectively probed with the use of the visual half-field paradigm. Based on responses to words presented in the dominant and non-dominant visual fields, the obtained laterality indices reveal differential involvement of the co-lateralized bilingual mechanisms in such a basic linguistic task as overt reading, depending on the number of languages a proficient bilingual uses in a given context. These results clearly indicate that one of the ways of obtaining highly proficient command of two or more languages is their functional separation at some intermediate level, whereby the lexical access is accompanied by weaker between-language interference. Thus, the adoption of a paradigm similar to the one used here opens a promising avenue for future research aimed at investigating the control mechanisms involved in the context-dependent utilization of linguistic skills in bilingual and multilingual individuals.

## Author contributions

This project was conceptualized by MK and GK. Data was collected by MK and BM, analyzed by MK, GK, BM, and JK, and interpreted by all the authors. The manuscript was written by GK, BM, JK, and MK.

### Conflict of interest statement

The authors declare that the research was conducted in the absence of any commercial or financial relationships that could be construed as a potential conflict of interest.
