# Supplementary material for: Co-lateralized bilingual mechanisms for reading in single and dual language contexts: evidence from visual half-field processing of action words in proficient bilinguals
Source: Front Psychol. 2015 Aug 7;6:1159. doi: 10.3389/fpsyg.2015.01159 (PMC4528090; doi:10.3389/fpsyg.2015.01159)
Supplement: Supplementary file 1 [file DataSheet1.DOCX]

**Appendix 1. List of words used in the experiment**

| Number of letters | Words in Polish | Words in English |
| --- | --- | --- |
| 3 | *kuć* (to forge) | row |
| 4 | *szyć* (to sew) | chop, fill, peel |
| 5 | *dziać* (to knit)*, kosić* (to mowe)*, kroić* (to slice)*, rąbać* (to chop)*, pisać* (to write) | close, shear |
| 6 | *czesać* (to comb)*, mielić* (to mill)*, strzyc* (to shear)*, zmywać* (to wash) | aiming, buckle, fasten, mixing, mowing, sawing, sewing, wiping |
| 7 | *celować* (to aim)*, czyścić* (to clean)*, malować* (to paint)*, masować* (to massage)*, mieszać* (to mix)*, obierać* (to peel)*, pakować* (to package)*, piłować* (to saw)*, rysować* (to draw)*, wiercić* (to drill)*, wycinać* (to cut)*, zamykać* (to close)*, zapinać* (to fasten) | combing, cutting, drawing, forging, ironing, milling, slicing, washing, writing |
| 8 | *odkurzać* (to vacuum)*, podlewać* (to water)*, prasować* (to iron)*, strzelać (*to shoot)*, szorować* (to scrub)*, wycierać* (to wipe)*, zamiatać* (to sweep) | brushing, cleaning, drilling, knitting, grinding, painting, screwing, shooting, sweeping, watering |
| 9 | *polerować* (to polish)*, szlifować* (to grind)*, wiosłować* (to row) | highlight, massaging, packaging, polishing, scrubbing, vacuuming |
| 10 | *podkreślać* (to highlight)*, przykręcać* (to screw)*, umocowywać* (to buckle) | photograph |
| 11 | *szczotkować* (to brush)*, szpachlować* (to fill) | - |
| 12 | *fotografować* (to photograph) | - |
